# Comparison of various microbial inocula for the efficient anaerobic digestion of *Laminaria hyperborea*

**DOI:** 10.1186/1472-6750-14-7

**Published:** 2014-01-23

**Authors:** Alastair D Sutherland, Joao C Varela

**Affiliations:** 1 Department of Biological and Biomedical Sciences, School of Health and Life Sciences, Glasgow Caledonian University, Cowcaddens Road, Glasgow G4 0BA, Scotland, UK; 2 Centre of Marine Sciences, University of Algarve, Campus de Gambelas, 8005-139, Faro, Portugal

**Keywords:** Seaweed, Microbial inoculum, Anaerobic digestion, *Laminaria hyperborea*, Methane

## Abstract

**Background:**

The hydrolysis of seaweed polysaccharides is the rate limiting step in anaerobic digestion (AD) of seaweeds. Seven different microbial inocula and a mixture of these (inoculum 8) were therefore compared in triplicate, each grown over four weeks in static culture for the ability to degrade *Laminaria hyperborea* seaweed and produce methane through AD.

**Results:**

All the inocula could degrade *L. hyperborea* and produce methane to some extent. However, an inoculum of slurry from a human sewage anaerobic digester, one of rumen contents from seaweed-eating North Ronaldsay sheep and inoculum 8 used most seaweed volatile solids (VS) (means ranged between 59 and 68% used), suggesting that these each had efficient seaweed polysaccharide digesting bacteria. The human sewage inoculum, an inoculum of anaerobic marine mud mixed with rotting seaweed and inoculum 8 all developed to give higher volumes of methane (means between 41 and 62.5 ml g^-1^ of seaweed VS by week four) ,compared to other inocula (means between 3.5 and 27.5 ml g^-1^ VS). Inoculum 8 also gave the highest acetate production (6.5 mmol g^-1^ VS) in a single-stage fermenter AD system and produced most methane (8.4 mL mmol acetate^-1^) in phase II of a two-stage AD system.

**Conclusions:**

Overall inoculum 8 was found to be the most efficient inoculum for AD of seaweed. The study therefore showed that selection and inclusion of efficient polysaccharide hydrolysing bacteria and methanogenic archaea in an inoculum offer increased methane productivity in AD of *L. hyperborea*. This inoculum will now being tested in larger scale (10L) continuously stirred reactors optimised for feed rate and retention time to determine maximum methane production under single-stage and two-stage AD systems.

## Background

There is a renewed interest in seaweeds as potential biomass for sustainable biofuel production; particularly as their growth does not compete for agricultural land or require water for irrigation. From the 1970s through to 1990s various groups were involved in assessing the suitability of seaweed (particularly *Macrocystis pyrifera*) as a biomass for anaerobic digestion (AD) to produce methane biofuel (see review [1]). The overall result of these studies indicated that seaweeds were generally a suitable biomass for AD.

In the Northern Atlantic *Laminaria*, *Fucus* and *Ascophyllum* species are particularly common brown algae found growing on the littoral and sub-littoral seashore. Few studies have examined AD of these seaweeds but Moen *et al*. [2,3] successfully demonstrated AD of *Laminaria hyperborea* and *Ascophyllum nodosum*, respectively*.* The efficient hydrolysis of seaweed polysaccharides, particularly alginates, was however seen as a rate limiting step for efficient AD to proceed [2,4,5]. Animal manure slurry is often utilised as an inoculum for anaerobic digesters, but various reports have suggested that specific bacteria capable of fermenting marine phycocolloids may accelerate and increase biogas production from seaweeds [reviewed in 4]. Williams *et al*. [6] studied the ruminal microbiota of the North Ronaldsay breed of sheep (the male adults of which survive almost entirely on a seaweed diet on the remote island of North Ronaldsay in the Orkney Islands, Scotland) and isolated bacteria and crude enzyme extracts that hydrolysed seaweed polysaccharides including laminarin, alginate, carrageenan, cellulose and to a lesser extent fucoidan. Additionally, in serial culture this rumen microbiota hydrolysed a range of seaweed polysaccharides and homogenates of *L. hyperborea*, mixed *Fucus* spp. and *A. nodosum* to produce methane and acetate. This particular rumen microbiota and bacterial isolates were therefore considered to represent potential adjunct organisms or enzymes which may improve hydrolysis of seaweed components and thus improve the efficiency of seaweed anaerobic digestion for methane biofuel production.

With a long term aim of developing and characterising an optimal inoculum with which to degrade seaweeds in AD, we compared the ability of a range of microbial inocula, including North Ronaldsay sheep rumen microbiota [6], to effectively hydrolyse *L. hyperborea* and produce methane biofuel. We examined these inocula in single-stage and two-stage fermenters in order to select the most productive inoculum for future optimisation studies in large scale (10L) fermenter systems. We studied single and two-stage systems because others have recommended [7] that seaweed AD is better conducted in two stages. In the first stage complex polysaccharide hydrolysis and acidogenesis to volatile fatty acids (VFAs) by fast growing and low pH tolerant bacteria is encouraged. The VFAs produced in the leachate of such stage one fermenters occurs at pH 5.5 to 6.0. The leachate is then neutralised and fed into phase II fermenters where acetate is then further converted to methane by acetoclastic methanogens. This two-stage system has been recommended for seaweed AD because seaweeds have a high sugar content and so tend to lower the fermenter pH with rapid growth of acidogenic bacteria, which may inhibit methanogenic archaea [7,8]. We aimed to select the inoculum which was most efficient in both systems.

## Results

### Comparison of AD inocula in single-stage fermenters

**Figure 1:**
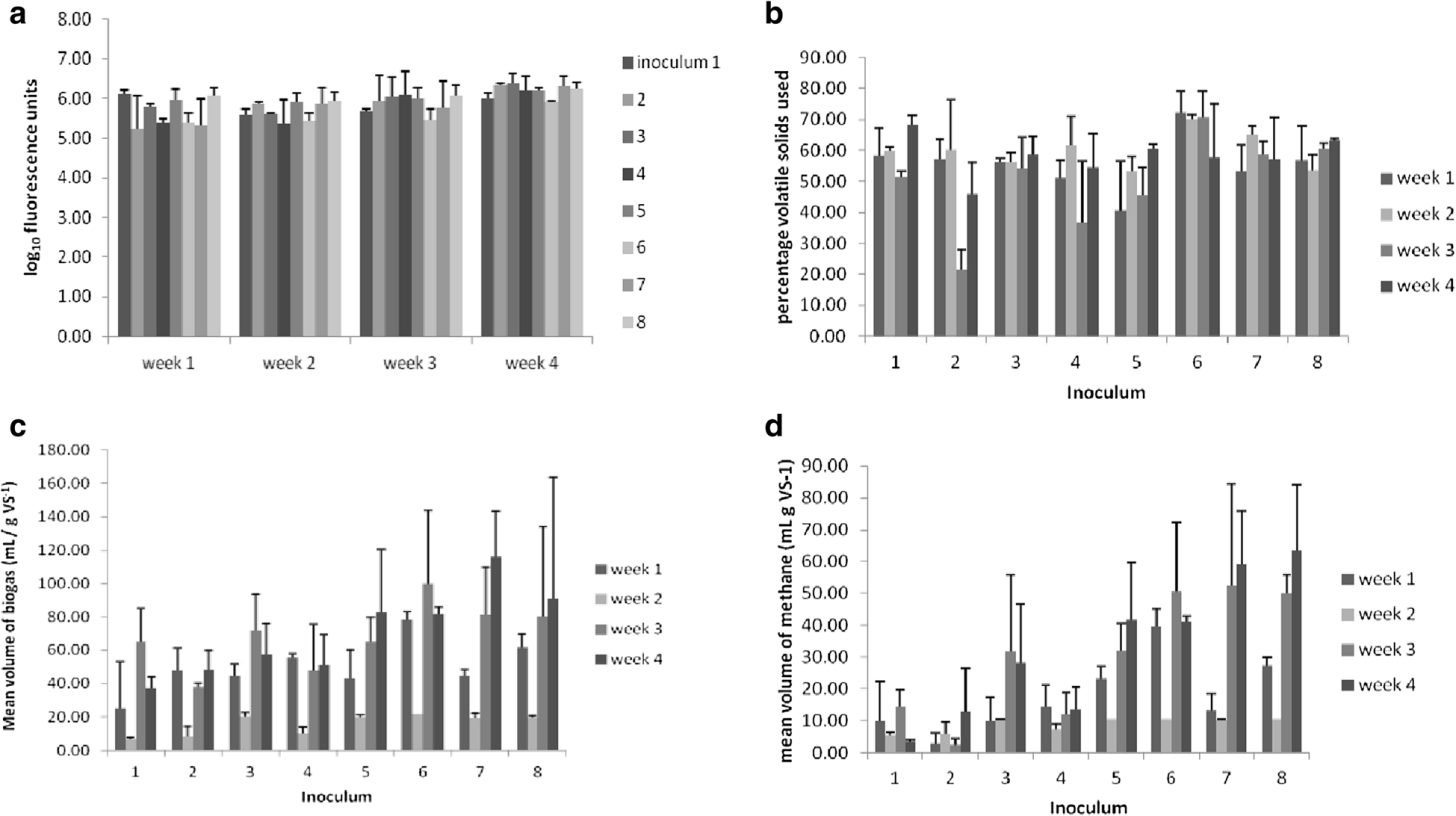
Operational parameters in single-stage fermenters (a) microbial viability, (b) utilisation of volatile solids, (c) biogas produced and (d) methane produced.

All the microbial inocula (for a description of the inocula see the Methods section) grew to give similar metabolic activity of around 6.0 log_10_ fluorescence units (Figure 1a) and so they clearly adapted to survive during *in vitro* fermentation of *L. hyperborea* using the mini-fermenters described in the methods section.

The *L. hyperborea* seaweed used in this study was added to fermenters as wet weight per volume. Samples of this feed were examined for the percentage total solids and volatile solids (VS) on three separate occasions giving a mean (±SD) percentage of 23.96 (± 2.32) TS and 20.13 (±0,98) VS, therefore showing the ability to reproducibly supply the same amount of wet weight feed to each fermenter.

All the inocula tested could hydrolyse *L. hyperborea* to some extent, but between 28 and 55% of VS were still undigested in individual fermenters. Figure 1b shows the mean percentage of VS used in triplicate fermenters of the same inoculum. Over the 4 week period Inoculum 6 gave the highest overall utilisation of seaweed VS (mean of 67.74% used) and this was significantly higher (*p* < 0.05) usage than by inocula 2, 3, 4, 5, and 7 (range 46.14% to 56.37%) but not by inocula 1 or 8 with means of 59.50 and 58.69% VS used, respectively.

Triplicate fermenters of each inoculum (1–8) were tested randomly on separate occasions. So it was interesting that all showed decreased biogas production in week 2 (various dates) and then recovered (Figure 1c). Thereafter, all inocula generally produced biogas above initial (week 1) biogas production levels. Inocula 5, 6, 7 and 8 (either from anaerobic digesters or a mixture of all inocula) had developed to give more biogas (mean of 82.0, 81.25, 115.42 and 72.08 mL g^-1^ VS, respectively) than other ruminant derived inocula by week 4. The percentage of methane and carbon dioxide in the biogas was analysed by GC and the volume of methane in biogas calculated from this. All inocula were capable of producing methane from seaweed (Figure 1d) but some gave significantly better yield than others. Inocula 5, 6, 7 and 8 had developed by week 4 to give higher volumes of methane production than other inocula (mean (± SD) of 41.25 (±18.01), 40.76 (±1.84), 58.55 (±16.81) and 63.10 (±20,40) ml g^-1^ VS, respectively). Inoculum 8 was highest but only significantly higher than inocula 1 to 5 [*p* < 0.05], not 6 or 7. There was a drop in methane production by most inocula in week 2, which coincides with the decrease in total biogas produced (Figure 1c).

Acetate production in fermenters over the 4 weeks was significantly greater by inoculum 1 (mean of 0.298 g/L) than by inocula 2, 3, 4, 6 (*p* < 0.05) and 7 (*p* < 0.01) but not inoculum 5 or 8. Inoculum 8 contained microbiota from inoculum 1 and so this is perhaps not surprising. Residual acetate from a single-stage fermenter can be converted to methane by acetoclastic methanogens in a second stage fermenter [7]. The calculated potential methane yield from acetate (mean results of three fermenters per inoculum sampled over four weeks) in the single-stage fermenters is given in Table 1. The theoretical methane production from acetate was calculated as 1 mole of acetate is converted into 1 mole of methane; and 1 mole of any gas at standard laboratory conditions (25°C, 1 atmosphere pressure) equals 22.47 L of gas.

**Table 1:** Calculated total methane yield from acetate and actual methane produced in single-stage fermenters

	**Inoculum**							
	**1**	**2**	**3**	**4**	**5**	**6**	**7**	**8**
Acetate* produced (mmol g^-1^ VS)	7.2	4.5	5.3	4.3	5.4	2.9	2.8	6.5
Calculated methane yield from acetate (litres g^-1^ VS)	0.161	0.101	0.120	0.097	0.122	0.066	0.064	0.145
Calculated total methane yield (litres g^-1^ VS)	0.185	0.118	0.177	0.130	0.199	0.167	0.160	0.253

*acetate produced is the mean cumulative amount over four weeks from triplicate single-stage fermenters. The total methane yield is the calculated methane yield from acetate added to the actual methane produced in the same fermenter over four weeks.

Taking the actual methane produced and combining it with the calculated methane yield from acetate (Table 1). Inoculum 8 gave the highest mean overall calculated methane yield (0.253 L g^-1^ VS), followed by inoculum 5, then inoculum 1 (0.199 L g^-1^ VS, 0.185 L g^-1^ VS, respectively).

### Phase I fermenter operation

Even running at a pH adjusted daily with 2M NaOH to be between 5.5 and 6.0 ( the range of pH the culture covered during daily fermentation was between 4.96 and 5.88) there was a substantial percentage of methane detected in the headspace biogas most weeks; with 34.80/65.11; 0.54/99.35 and 42.13/57.72 % CH_4_/CO_2_ in weeks 2,3 and 4, respectively (the week 1 gas sample was lost due to human error). The volume of biogas was not measured in this study. That methane was produced at such low fermenter pH was in agreement with the study of Vergara-Fernández *et al. *[7] who also detected methane gas production in a phase 1 fermenter for seaweed hydrolysis. The growth of inoculum 6 used in the phase I fermenter was stable over the 4 week period with log_10_ fluorescein diacetate (FDA) values of 5.76, 5.72, 5.68 and 5.37 for weeks 1 to 4, respectively; and so showed good survival of the inoculum under the phase I fermenter conditions. The % of total solids (TS) used over the 4 week fermentation was 40.00, 50.78, 60.75 and 62.00%, respectively; which was, at least initially, less than the utilisation by inoculum 6 during 4 weeks of the single-stage fermentations where there was an average of 67% usage (see above). The lower VS usage in the first two weeks of fermentation may indicate that the hydrolytic bacteria had to adapt to the low fermenter pH.

The acetate concentration in the pooled phase I fermenter leachate was 0.518 g/L. This supplied 3.5 mmol of acetate to each phase II fermenter over 4 changes of leachate (400 mL) which theoretically could have given 22.17 mL of methane.

### Comparison of inocula in phase II fermenters

The volume of biogas (expressed as mL mmol^-^1 acetate) produced over four weeks from 400 mL of leachate (3.5 mM of acetate) generated in a phase I fermenter (see Methods section); the percentage of methane in that biogas and the total methane production in mL mmol^-1^ acetate over four weeks for each inoculum is shown in Table 2. Inoculum 8 produced most methane (8.4 mL mmol^-1^ acetate), followed by inoculum 6 (7.31mL mmol^-1^ acetate). Other inocula gave less than 3.0 mL mmol^-1^ acetate of methane each and inocula 2, 3 4 and 7 had particularly low methane levels.

**Table 2:** Biogas and methane production from phase II fermenters fed with phase I fermenter leachate

	**Inoculum**							
	**1**	**2**	**3**	**4**	**5**	**6**	**7**	**8**
Mean biogas* (mL mmol^-1^ acetate)	10.0	12.9	21.4	17.1	14.3	22.9	12.9	22.9
Mean % methane	25.35	0.70	5.40	0.16	18.15	31.98	1.70	36.88
Mean methane volume produced (mL mmol^-1^ acetate)	2.3	0.5	0.6	0.1	0.6	7.3	0.1	8.4

*The volume of biogas (expressed as mL mmol^-1^ acetate) produced over four weeks from 400 mL of leachate (3.5 mM of acetate) generated in a phase I fermenter.

## Discussion

All the inocula tested could hydrolyse *L. hyperborea* to some extent (Figure 1b) but even by week 4, inoculum 6, 1 and 8, the most hydrolytic inocula, still had means of 32.26, 40.50 and 41.31% VS remaining, respectively. It will be interesting to determine in further fermenter studies what the composition of this residue is. It possibly contains a high percentage of alginates since Moen *et al. *[2] reported that between 13 and 50% of alginates were resistant to AD, depending upon levels of *L. hyperborea* polyphenols present and the ratio of calcium and sodium alginate salt forms. The high utilisation of VS by inoculum 6 (human sewage AD leachate) and inoculum 1 (North Ronaldsay sheep rumen microbiota) suggests that they had efficient seaweed polysaccharide hydrolysing bacteria. Moreover, this result is in agreement with preliminary studies (A.D. Sutherland, unreported results) in which it was found that a human sewage AD effluent recovered in Scotland was similarly most effective in AD of *L. hyperborea*; this was thus not a unique occurrence by the Portuguese sourced inoculum 6. Inoculum 1 was previously shown to have bacteria producing polysaccharidases capable of hydrolysing seaweed polysaccharides [6] and Hehemann *et al. *[9] have recently reported the presence of alginate lyase genes in human microbiota which have been derived from marine bacteria. Alginates and other seaweed polysaccharides are used as thickeners in foods, toothpastes and cosmetics and it is conceivable that human gut bacteria with hydrolytic enzymes survive in human sewage digestate (inoculum 6). Therefore, both inoculum 6 and 1 may offer adjunct bacteria or enzymes to improve the efficiency of hydrolysis and are likely to have contributed to the high utilisation of VS by inoculum 8 (a mixture of all the inocula studied).

Since there was no concomitant reduction in the %VS used (Figure 1b) the drop in biogas and methane production seen in all week 2 samples may have been due to an initial reduction in methanogenic archaea. These are very slow growing and very strict anaerobes, the decrease of which may have been overcome by biofilm formation that was observed to have developed on the fermenter walls by week 3.

Since inoculum 8 gave the highest levels of methane production by week 4 it would be advantageous to use this mixed inoculum for AD of *L. hyperborea.* It would be interesting, in further studies, to add selected bacterial isolates rather than the whole rumen microbiota (e.g. alginate lyase producers as isolated by Williams *et al*. [6]) to inoculum 6 to see if it improves VS usage. Also, inoculum 6 should be investigated to determine what endogenous alginate hydrolysers it may possess.

That inoculum 1 had high levels of acetate may be the reason why this inoculum (and to some degree inoculum 3 [normal sheep rumen microbiota]) required most pH adjustment, reaching lows of 5.8 within 24 hours of adjustment. This may also be a reason why methanogenesis was progressively reduced in some replicates of these inocula, since methanogens grow poorly at pH less than 6.0 and would possibly have begun to be washed out of the semi-continuous fermenter. Even without methane production, as long as the microbiota in an inoculum can degrade seaweed solids to produce VFAs, these may be utilisable for biofuel and other valuable chemical feedstock production via the carboxylate platform [10], where lack of methane production is desirable. One example of this would be inoculum 1, which could be a valuable source of hydrolytic bacteria for the latter platform. If the hydraulic retention time in a single-stage fermenter is too short to allow all acetate produced to be converted to methane by acetoclastic methanogens then accumulated acetate can be fed into a second (phase II) fermenter with immobilised acetoclastic methanogens [7].

Inoculum 8 gave the highest methane production when considering actual methane produced plus the calculated methane production from acetate (Table 1). This further showed that inoculum 8 was the most productive inoculum in single-stage fermenters.

A two-stage fermenter system was operated in which inoculum 6 was used to produce acetate in a phase I fermenter. The leachate of the latter was then fed into individual phase II fermenters containing inocula 1 to 8 in order to determine which inoculum was most productive in acetoclastic methanogenesis.

Inocula 2, 3, 4 and 7 gave poor methane production from acetate in the second phase of a two-stage fermenter system, indicating they do not have sufficient levels of acetoclastic methanogens for operation of a phase II fermenter. Levels of acetoclastic methanogens in normal sheep rumen are for example very low; however, acetoclastic methanogens such as *Methanosarcina barkeri* are present and can grow to significant levels if dietary changes such as high starch content occurs [11]. The results for the phase II fermenters complement the findings for the single stage fermenters where again inocula 8 and 6 were the most efficient inocula and inoculum 8 the best at producing methane from acetate (8.4 mL methane mMol acetate^-1^). Inoculum 1 was the third best acetoclastic methanogenic inoculum (Table 2) and it will be interesting to determine by metagenomics what archaea are present in inocula 8, 6 and 1 after growth in a phase II fermenter.

In general, laboratory studies have found that methane yield is greater from brown algae than from green algae [4]. There are very few studies conducted on AD of *Laminaria* sp. but Hanssen *et al*. [12] reported only a 36% usage of VS and 280 mL methane gVS^-1^ in an 8L fermentation volume with a retention time of 24 days. More recently Matsui *et al*. [13] using a separated acidic and methanogenic phase commercial scale AD plant of 30M^3^ reported 22 mL methane g^-1^ wet weight seaweed. In the present study the maximum % of VS used in any fermenter using inoculum 8 (the most productive inoculum) was 66.54% and a yield of 83 mL methane g^-1^ VS(17 mL methane g^-1^ wet weight seaweed). However, the present study aimed to compare the ability of several inocula to ferment *L.* hyperborea under conditions of low feed rate (1% wet weight *L.* hyperborea day^-1^) and high turnover of the liquid phase (a complete change in leachate week^-1^) in order to minimise the effects of acidification and product feedback inhibition previously noted by others [7,13]. The study was not conducted under optimised AD conditions, but was designed to compare several inocula in one study to select the most efficient in the system used. The methane yield by inoculum 8 will likely be increased in a large scale (10 L) continuously stirred fermenter optimised for feed rate, solids and liquids retention time and automatic pH adjustment; and this study is now intended by this group. As hydrolysis of polysaccharides is the rate limiting step in seaweed AD, the need for more effective inocula for AD of brown seaweed is reflected in the conclusions of others [2-4]. Wargacki *et al*. [8] to this end have recently cloned alginate lyase genes into *E. coli* and have produced bioethanol from seaweed at near 80% of the theoretical yield. Like others [14-16] who found marine sediments were efficient inocula in seaweed AD we adopted, instead of gene cloning, to study complex microbiota in inocula from diverse locations in order to select efficient seaweed hydrolysers for AD, and found that a mixture of all the inocula (including North Ronaldsay sheep rumen and human sewage AD slurry microbiota) was most productive.

We are currently employing dissociative gradient gel electrophoresis and next generation sequencing on PCR amplified 16S rRNA genes to characterise the microbial consortia present in the various inocula studied here. DNA was also extracted from each fermenter weekly and metagenomics will be used to examine the temporal and spatial development of microbial consortia. This may indicate, for instance, which microbes from the various inocula developed in inoculum 8 to make it the most efficient in methane production. It may indicate why methane production dropped in week 2 in all fermenters and it may also establish which hydrolytic bacteria and methanogenic archaea were active in single-stage and two-stage (phase I and II) fermenters.

## Conclusions

Several microbial inoculum were examined for the ability to hydrolyse polysaccharides and produce methane in AD of *L. hyperborea*. All the inocula could hydrolyse *L. hyperborea* and produce methane to some extent but there were significant differences in the %VS used and the amount of methane produced thus showing that selection of an inoculum can improve AD. Inoculum 8, which corresponded to a mixture of inocula 1–7, proved to be highly hydrolytic and produced the most methane after AD in a single-stage (0.253 Litres methane g^-1^ VS when including calculated methane from acetate)or in phase II of a two-stage digester system (8.4 mL methane mmol^-1^ acetate) (Tables 1 and 2, respectively). This showed that careful selection of a complex microbial inoculum can increase the efficiency of seaweed AD, most likely due to combining effective polysaccharolytic bacteria, in this instance from North Ronaldsay sheep rumen microbiota [6], with appropriate methanogenic archaea which have developed in AD reactors, as in inoculum 6. When examined individually, normal (grass fed) sheep rumen microbiota (inoculum 4) did not perform as well and neither did faecal microbiota from North Ronaldsay sheep or normal sheep (Tables 1 and 2). This group will now use inoculum 8 in optimised single stage and two stage AD systems to determine methane productivity.

## Methods

Throughout the study all materials and reagents used were pre-reduced by gassing in a sealed plastic bag flushed with a mixture of 80% nitrogen, 10% carbon dioxide, 10% hydrogen in the presence of a palladium catalyst (Don Whitley, Scientific) to convert oxygen to water. Strict anaerobic conditions were observed by the reduction of a resazurin based indicator strip (Oxoid, UK) from pink to colourless.

### Inoculum collection

Samples of the various microbial inocula for AD had been either, collected in Scotland in 100 mL aliquots (completely filled bottles) and frozen and transported by courier at -20°C, which was previously shown to retain microbial viability in these samples [6] or were collected in The Algarve, Portugal and frozen on collection. The inocula were:

1. North Ronaldsay sheep rumen contents, obtained at slaughter in Kirkwell, Scotland.

2. North Ronaldsay sheep faeces, obtained at slaughter in Kirkwell, Scotland.

3. Normal sheep (grass eating) rumen contents, obtained at slaughter in Paisley, Scotland

4. Normal sheep (grass eating) faeces, obtained at slaughter in Paisley, Scotland.

5. A mixture of municipal AD fermenter leachates kindly supplied by Biogen Greenfinch, UK.

6. Human sewage AD leachate kindly supplied by ETAR, Lagos, Portugal.

7. Marine sediments (anaerobic, black mud collected from the Ria de Formosa, Faro, Portugal and mixed with naturally rotting *L. hyperborea* which was anaerobically fermented for 48 hours after collection.

8. A mixture of all the above inocula (in equal proportions).

#### *L. hyperborea preparation*

Frozen *L. hyperborea* collected as beach cast seaweed (having no obvious signs of deterioration compared to fresh seaweed) on the Firth of Forth, near Edinburgh, Scotland was stored at -20°C upon courier delivery in frozen condition.

The seaweed was thawed and washed under tap water to remove sand and dirt. The seaweed fronds and stipe (5 Kg) were ground with a kitchen mincer (6 mm diameter holes) and then were thoroughly mixed by hand to obtain a homogeneous mass. Aliquots (100 g) were frozen at -20°C, defrosted as required and again mixed thoroughly by hand. Amounts of 12 g were weighed and reduced to approximately 2 mm diameter particles in 60 mL of phosphate buffered saline (PBS) using a Braun kitchen electric hand mixer with a grinding bowl. The blended seaweed (20% wet weight/volume [w/v]) was autoclaved, gassed under nitrogen and then stored at 4°C and used within 1 week.

### Single-stage AD fermenter operation

**Figure 2:**
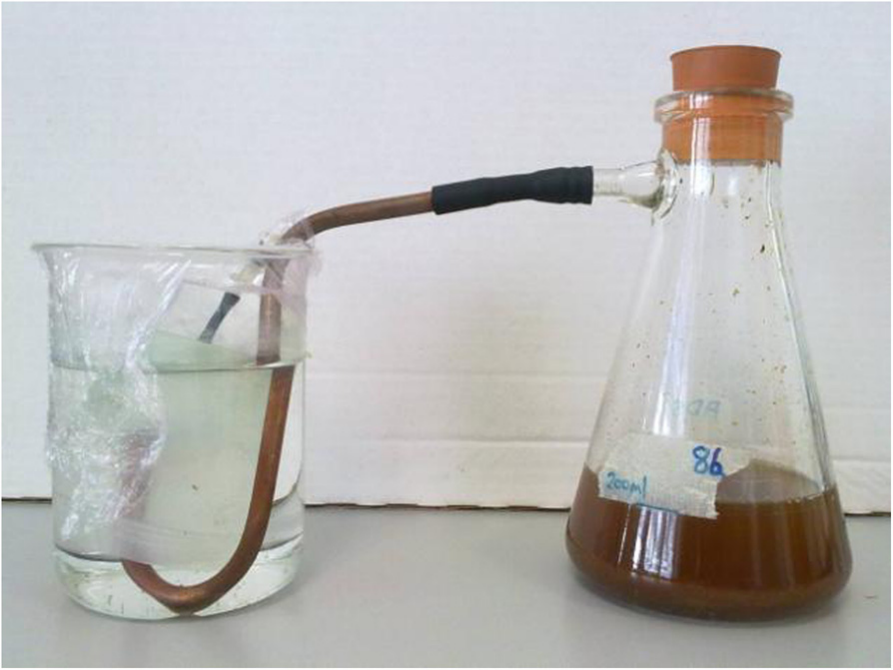
Conical-flask fermenter with gas collector on side-arm.

Each inoculum was tested randomly on three separate occasions (up to 10 fermenters were run at a time, the whole experiment taking three months to complete). Because the inocula had been frozen upon collection, 100-mL aliquots of each were defrosted at 30°C and then immediately placed under anaerobic conditions into sterile pre-reduced 500 mL Duran bottles. Each bottle contained 300 mL of phosphate buffered saline (PBS) supplemented with 0.5 mL each of 20% (w/v) sterile glucose and yeast extract. Incubation was carried out for 48 h at 37°C. Each sample was then split into 4 x100 mL volumes: one volume (avoiding any debris from the original inoculum) was put into a sterile pre-reduced conical-flask fermenter with a water-displacement gas collector (Figure 2) and three were frozen again for further studies. To the fermenters were added 10 mL of 20% (w/v) *L. hyperborea* in PBS and 90 mL of PBS to give a final seaweed concentration of 1.0% (w/v) in a 200 mL volume (this was day 1 week 1 for each fermenter). The samples were adjusted to pH 7.0 with 2M Na OH as required and then incubated at 37°C. These samples were then treated in triplicate semi-continuous culture as in Table 3 for 4 weeks each (4 hydraulic retention times [HRT]).

**Table 3:** **Weekly protocol for the semi-continuous fermentation of *L. hyperborea *** **in single-stage fermenters**

**Treatment**			**Day**				
	1*	**2**	**3**	**4**	**5**	**6**	**7**
	**Mon**	**Tue**	**Wed**	**Thu**	**Fri**	**Sat**	**Sun**
Volume of leachate removed (mL)	200	0	60	0	60	0	0
	(140 mL returned)						
Volume of 20% wet W/V *L. hyperborea* added	10	10	10	10	20	0	0
Volume of PBS added	50	0	50	0	40	0	0
Seaweed removed	yes	no	no	no	no	no	no

*From week two onwards, on day 1 of each week the contents of each fermenter were removed to a sterile plastic pot. A 140 mL volume of the leachate of each fermenter was poured back into the fermenter, 10 mL of *L. hyperborea* seaweed was added and the fermenter volume was made up to 200 mL with PBS. In this manner the fermenter volume was maintained at 200 mL; seaweed was added daily and leachate was removed every second day. The residual seaweed was dried for estimation of residual total solids, then ash and volatile solids (see Methods section). This semi-continuous system was designed to reduce acidity and product feedback inhibition from volatile fatty acids, whilst having a long enough hydraulic retention time to allow maintenance of slow growing and acid sensitive methanogens and also allow digestion of seaweed to occur and was similar to the phase 1 system of a two-stage semi-continuous system adopted by Vergara-Fernández *et al.*[7].

The fermenter leachates were examined every 24 hours for pH (which was adjusted to pH >6.5 as required with 2M NaOH). Weekly, the microbial metabolic activity (FDA fluorescence), the volume of biogas produced and the biogas composition on a gas chromatograph (% methane and carbon dioxide) was measured. The FDA assay was carried out essentially as published [17]. A 1/50 dilution of FDA stock solution (10 mg/mL in acetone) was made in 100 mM MOPS buffer (pH 7.0). Volumes (100 μL) of this were added to duplicate 100 μL volumes of each inoculum sample (at 10^-1^ and 10^-2^ dilution) in black microtitre plates. Samples were incubated for 1 hour and fluorescence read (emission 494 and excitation 520 nm wavelength) after subtraction of the reading of a blank of 100 μL of PBS plus FDA. The fluorescent reading was multiplied by the reciprocal of the dilution factor of the inoculum and then converted to log_10_. The biogas composition was determined on a gas chromatograph, as previously described [6].

Also, weekly, TS, ash and VS content of residual seaweed in each fermenter were measured by drying the samples at 100°C overnight and weighing (to obtain the TS) and then further drying at 530°C for approximately 6 hours, until there was no further weight loss and weighing again to get ash content (and by subtraction from the TS, the VS content). These figures were compared with the TS, VS and ash content of the same weight of original seaweed to calculate the percentage of each that were removed by AD. A compositional analysis of *Laminaria* sp. has been carried out previously by others [2,4,12,13]. Each fermenter received 60 mL of 20% (w/v) seaweed per week; which equals 12 g of wet weight seaweed (2.4 g VS).

Acetate in the fermenter leachates was measured by an enzymatic kit (BioPharm Ltd.) as previously described [6].

### Comparison of AD inocula in a two-stage semi-continuous fermenter system

The same inocula as were compared in the single-stage AD fermenters (see above) were also compared in a two-stage (phase I and II) system to determine which inoculum was optimum for methanogenesis in phase II. To make the stage II system fermenters comparable they were all fed from a large volume of stored leachate as feedstock derived from a phase I fermenter run with inoculum 6, which had the best usage of *L. hyperborea* VS in the single-stage system.

### Production of acetate in a semi-continuous phase I AD fermenter

A 100 mL volume of frozen inoculum 6 was defrosted and added to 50 mL of sterile 20% wet weight per volume (w/v) *L. hyperborea* seaweed and 850 mL of sterile distilled water in a pre-reduced one litre Dewar bottle as a fermenter vessel. The culture was adjusted to pH 5.5-6.0 by the addition of 2M NaOH to encourage growth of hydrolytic and acetogenic bacteria. The fermenter therefore had a volume 5-fold higher than that used in single-stage fermentations (see above). The fermenter was then run semi-continuously in the same manner as in Table 3 but with all volumes increased 5-fold. The residual seaweed VS was determined weekly and headspace gases were monitored for methane production. The fermenter was run for 4 weeks with 1 litre of leachate collected weekly, pooled and frozen at -20°C until required. The pooled leachate was defrosted, the pH adjusted to 8.0 with NaOH to convert VFAs to their less volatile salt form and the leachate was then centrifuged at 3,000 × *g* for 30 min to pellet bacteria and the supernatant autoclaved to sterilise it and the pH readjusted to 7.0 and stored at 4°C until used.

### Production of methane in semi-continuous phase II AD fermenters

A 100 mL volume of each frozen inoculum (1 to 8) was defrosted and resuscitated with glucose and yeast extract (as for the single-stage fermenters above). A 100 mL volume of each inoculum was then added with 100 mL of phase I leachate as feedstock to sterilised, pre-reduced side-arm flask fermenters with gas collectors (Figure 2). The fermenters additionally had ¼ of a Scotch-Brite plastic scouring pad in them to encourage methanogens in particular to adhere to them and slow wash out. The volume of biogas produced and its composition as % methane/CO_2_ was measured and pH was monitored regularly. When biogas production ceased 100 mL of leachate was removed from a fermenter and was replaced with a further 100 mL of phase I fermenter leachate. The process was repeated until 4 cycles of leachate feeding had occurred.

### Statistical analysis

All comparisons of data from fermenters were done on SigmaPlot for Windows version 10, using a paired Student’s T-test.

## Competing interests

The authors declare that they have no competing interests

## Authors’ contributions

AS and JV were both involved in conception of the study, sample collection, manuscript writing and editing and interpretation of data. AS was responsible for fermenter operations and data analysis. All authors read and approved the final manuscript.

## Authors’ information

AS is a senior lecturer and researcher in microbiology at Glasgow Caledonian University. He held a Marie Curie senior researcher fellowship and was seconded to Centre of Marine Sciences, University of Algarve for two years during which the work in this manuscript was carried out. JV is a lecturer at the University of Algarve and the head of the MarBiotech research group of the Centre of Marine Sciences of Algarve (CCMAR).
